# Association Properties and Unfolding of a βγ-Crystallin Domain of a Vibrio-Specific Protein

**DOI:** 10.1371/journal.pone.0053610

**Published:** 2013-01-22

**Authors:** Shashi Kumar Suman, Daddali Ravindra, Yogendra Sharma, Amita Mishra

**Affiliations:** Centre for Cellular and Molecular Biology, Council of Scientific and Industrial Research, Uppal Road, Hyderabad, India; University of Michigan, United States of America

## Abstract

The βγ-crystallin superfamily possesses a large number of versatile members, of which only a few members other than lens βγ-crystallins have been studied. Understanding the non-crystallin functions as well as origin of crystallin-like properties of such proteins is possible by exploring novel members from diverse sources. We describe a novel βγ-crystallin domain with S-type (Spherulin 3a type) Greek key motifs in protein vibrillin from a pathogenic bacterium *Vibrio cholerae*. This domain is a part of a large *Vibrio*-specific protein prevalent in *Vibrio* species (found in at least fourteen different strains sequenced so far). The domain contains two canonical N/D-N/D-X-X-S/T-S Ca^2+^-binding motifs, and bind Ca^2+^. Unlike spherulin 3a and other microbial homologues studied so far, βγ-crystallin domain of vibrillin self-associates forming oligomers of various sizes including dimers. The fractionated dimers readily form octamers in concentration-dependent manner, suggesting an association between these two major forms. The domain associates/dissociates forming dimers at the cost of monomeric populations in the presence of Ca^2+^. No such effect of Ca^2+^ has been observed in oligomeric species. The equilibrium unfolding of both forms follows a similar pattern, with the formation of an unfolding intermediate at sub-molar concentrations of denaturant. These properties exhibited by this βγ-crystallin domain are not shown by any other domain studied so far, demonstrating the diversity in domain properties.

## Introduction

The surge in genome sequencing of diverse species continues to provide a wealth of uncoded information, on the features and functions of homologous proteins or domains. One of such domains is the βγ-crystallin domain found in proteins from micro-organisms to vertebrates [1]. A βγ-crystallin is a globular domain made up of two Greek key motifs, each containing eight β-strands as seen in the classical structure of lens protein, γ-crystallin [2], [3]. There are differences in the sequence of these domains from various proteins, except the presence of signature sequences of βγ-crystallins. In more diverse cases, it is even difficult to clearly identify a sequence as of βγ-crystallin domain due to high variations even in the signature sequence. Structurally, these domains have similar topology [4]–[8], and are thought to be designed as a stable domain during evolution [9]–[11]. While individual βγ-crystallin domains adopt stable β-sheet folded structure, a few rare cases, wherein a βγ-crystallin is intrinsically unstructured which folds upon binding Ca^2+^, have been identified [12], [13]. Presence of a βγ-domain in several pathogenic species arouses questions about their functions in these species. It has been predicted that Yersinia crystallin might play a role in the low Ca^2+^ response in this pathogen, as this domain is largely unstructured in the apo form, which refolds in the presence of Ca^2+^, thus implicating this protein in Ca^2+^-dependent virulency [12].

In our attempts to study more members from pathogenic species, we have selected a protein containing βγ-crystallin domain in the genome of *Vibrio cholerae*, using the sequence of Yersinia crystallin as template sequence. This hypothetical protein is named vibrillin. Due to the absence of some of the key signatures of Greek key motifs in vibrillin domain, the motifs were earlier classified as S-type motifs (on par with Spherulin 3a) [7] and not as A-type and B-type motifs of Protein S or lens γ-crystallin [14]. Our analysis suggests that vibrillin is a *Vibrio*-specific protein presents in many *Vibrio* species, but not in the genome of any other species till now. We report that this domain possesses diverse properties, not exhibited so far by any other βγ-crystallins, including Spherulin 3a. The domain undergoes dynamic self-association between monomeric, dimeric and oligomeric forms in concentration-dependent manner which is partially influenced by Ca^2+^. We provide extensive data on equilibrium unfolding of oligomeric (octameric) form that demonstrates the presence of an intermediate state before protein dissociates as monomer. Quaternary feature of intermediate state of dimeric form is different than that of octameric forms, as intermediate state of dimeric population associates into oligomers, whereas partially unfolded intermediate state of octameric vibrillin forms insoluble aggregates and Ca^2+^ assists in dissociation of the oligomeric protein to its dimeric form.

## Materials and Methods

### Cloning and over-expression of a βγ-crystallin domain from *Vibrio cholarae*

The genomic DNA of *Vibrio cholarae* was a kind gift from Dr. Rukhsana Chowdhury, IICB, Kolkata, India. The region of interest (173 to 256 amino acids) from a hypothetical protein (accession number NP_230470) of *Vibrio cholerae* was cloned in pET21a expression vector (Novagen) at *NdeI* and *Bam*HI sites. Vibrillin was over-expressed in the bacterial strain *E. coli* BL21(DE3) (Invitrogen) in LB medium after induction with 1 mM isopropyl thio-β-D-galactopyranoside (IPTG) for 10 h at 37°C.

### Purification of recombinant protein

Vibrillin formed inclusion bodies during over-expression. The protein was refolded from insoluble fraction using an on-column refolding procedure. Briefly, inclusion bodies, isolated by sonication and centrifugation of cell pellets, were further washed with 1 M urea and 0.1% CHAPS. Washed inclusion bodies were solubilized in 50 mM Tris buffer, pH 8.5, containing 3.5 M urea, 1 mM DTT (buffer A) and loaded on a pre-equilibrated Q-Sepharose column with buffer A. The protein was refolded by setting a gradient between buffer A and buffer A without urea. After this step, protein was eluted using a linear gradient of 0 M to 1.5 M NaCl. The fractions containing protein were concentrated by ultra-filtration using an Amicon cell with a 5 kDa molecular mass cut off membrane. The concentrated protein was finally purified by Superdex-75 gel filtration column on a Bio-Rad Duo-Flow purification system.

### Hydrodynamic volume

Hydrodynamic volumes of vibrillin were determined by gel filtration on a Superdex-75 (Wipro GE) column, calibrated using standard molecular mass markers. The gel filtration was performed in 50 mM Tris-Cl (pH 7.0), 100 mM KCl, containing either 1 mM EDTA or 5 mM Ca^2+^ at a flow rate of 0.4 ml/min.

### Glutaraldehyde cross-linking

Both forms of vibrillin (oligomeric and dimeric) (1 mg/ml) was incubated with 0.8% glutaraldehyde for 40 min. The reaction was stopped using 1 M Tris buffer, pH 7.5. SDS-gel loading buffer was added to the reaction samples, boiled and analysed on 15% SDS-PAGE calibrated with appropriate molecular mass markers. The protein bands were visualized by Coomassie Brilliant Blue R250 dye.

### Dynamic light scattering

Dynamic-light scattering measurements were performed on a SZ-100 Horiba instrument at 20°C. Approximately 0.5 mg/ml of protein was diluted in 50 mM Tris, pH 7.5, 100 mM KCL and centrifuged at 14,000 rpm for 5 minutes before placing into the cuvette.

### Analytical ultracentrifugation (AUC)

AUC was carried out on a Beckman Optima XL-I analytical ultracentifuge (Beckman Coulter, Fullerton, CA, USA) operating in sedimentation velocity mode with an aluminium centerpieces. Protein samples (0.5 mg/ml) prepared in 50 mM Tris pH 7.5, 100 mM KCl were centrifuged at a speed of 60,000 rpm at 20°C in a Ti60 rotor and data were collected using 280 nm absorbance optics for a total of 98 scans with the rate of one scan per every 6 minutes. The data were analyzed using the c(s) method in SedFit software to compute the molecular weight, from the AUC data using non-linear regression fitting of the sedimenting boundary profile with Lamm equation:
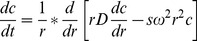
which describes the concentration distribution c(r, t) of a species with sedimentation coefficient s and diffusion coefficient D in a sector-shaped volume in the centrifugal field ω^2^r.

### Isothermal titration calorimetry (ITC)

Ca^2+^-binding to vibrillin was evaluated by ITC on a Microcal VP-ITC (Microcal Inc., USA). The protein solutions and CaCl_2_ (10 mM) were prepared in a Chelex-treated 50 mM Tris, pH 7.0 containing 100 mM KCl buffer. 1.4 ml of protein solution (100 µM) was used for the binding experiment at 30°C. Aliquots of 2 µl of CaCl_2_ as ligand solution were injected for each titration. Appropriate blank was obtained by titrating the buffer with identical concentrations of Ca^2+^. The curve fitting of the data after subtraction with appropriate buffer blank was performed using the software Origin (version 6.0) supplied by Microcal.

### Circular dichroism (CD) spectroscopy

Far- and near-UV CD spectra of vibrillin were recorded on a Jasco-815 spectropolarimeter using 0.01 cm and 1 cm path length cuvettes respectively at room temperature. The change in protein conformation upon binding Ca^2+^ was studied by titrating the protein solutions with aliquots of standard CaCl_2_ solution.

### Thermal unfolding

Thermal unfolding experiments were performed by monitoring the change in ellipticity in far-UV range (208, 216 or 218 nm) on a Jasco J-815 spectropolarimeter as a function of temperature at the rate of 1°C/min using a Jasco peltier system attached to the sample holder. Protein solution (0.1 mg/ml) in a 1 mm path length cuvette was used to monitor changes in ellipticity between 25°C to 85°C. Data obtained were plotted and best fitted using Origin 6.0.

### Fluorescence spectroscopy

Intrinsic fluorescence emission spectra of protein samples (0.1 mg/ml concentration) were recorded on a F-4500 Fluorescence spectrofluorimeter (Hitachi Inc., Japan) in 50 mM Tris-HCl, pH 7.0, 100 mM KCl. The spectra (300–450 nm) were recorded at an excitation wavelength of 295 nm in correct spectrum mode of the instrument using excitation and emission band passes of 5 nm each. The effect of Ca^2+^ was studied by titrating the protein solutions with standard aliquots of CaCl_2_ solution from 10 µM to 5 mM concentrations.

Change in surface hydrophobicity of protein samples in different concentrations of denaturant GdmCl (0 to 2 M) was monitored using bis-ANS (10 µM) as probe. The samples were excited at 395 nm, and emission spectra were recorded between 400 nm and 650 nm. The spectra were corrected for equal concentrations of bis-ANS in buffer.

### Equilibrium unfolding studies

Equilibrium unfolding of vibrillin was studied using guanidinium chloride (GdmCl) in the range of 0–6 M concentration with an interval of 0.1 M GdmCl (total of 60 data points for each unfolding transition) in the apo (Ca^2+^-free) and holo (Ca^2+^-bound form) states. Protein samples prepared in increasing concentrations of GdmCl (0 to 6 M) in 50 mM Tris buffer, pH 7.0, containing 100 mM KCl either with 1 mM EDTA or with 5 mM CaCl_2_, incubated for different times (2 to 48 hours) at room temperature, and Trp fluorescence emission spectra were recorded by exciting the samples at 295 nm and unfolding was monitored by following the changes in intrinsic Trp emission fluorescence. The ratios of fluorescence intensity (at 360/320 nm) was plotted as a function of GdmCl concentration and the data were fitted to the following equation, which relates fluorescence intensity to the extent of unfolding. The free energy of unfolding (ΔG_D_) determined from the ratio of native to denatured protein at each GdmCl concentration, was plotted against GdmCl concentration [15]. Equation used for two-state model:
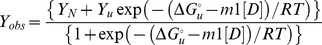
Where Y_N_ is signal of the folded protein, Y_u_ is signal of the unfolded forms, m is denaturant dependence of the free energy change in units of kcal mol^−1^ M^−1^, R is the universal gas constant (1.987 cal mol^−1^ K^−1^) and T is the absolute temperature.

## Results

### Vibrillin is a *Vibrio* species specific βγ-crystallin

**Figure 1:**
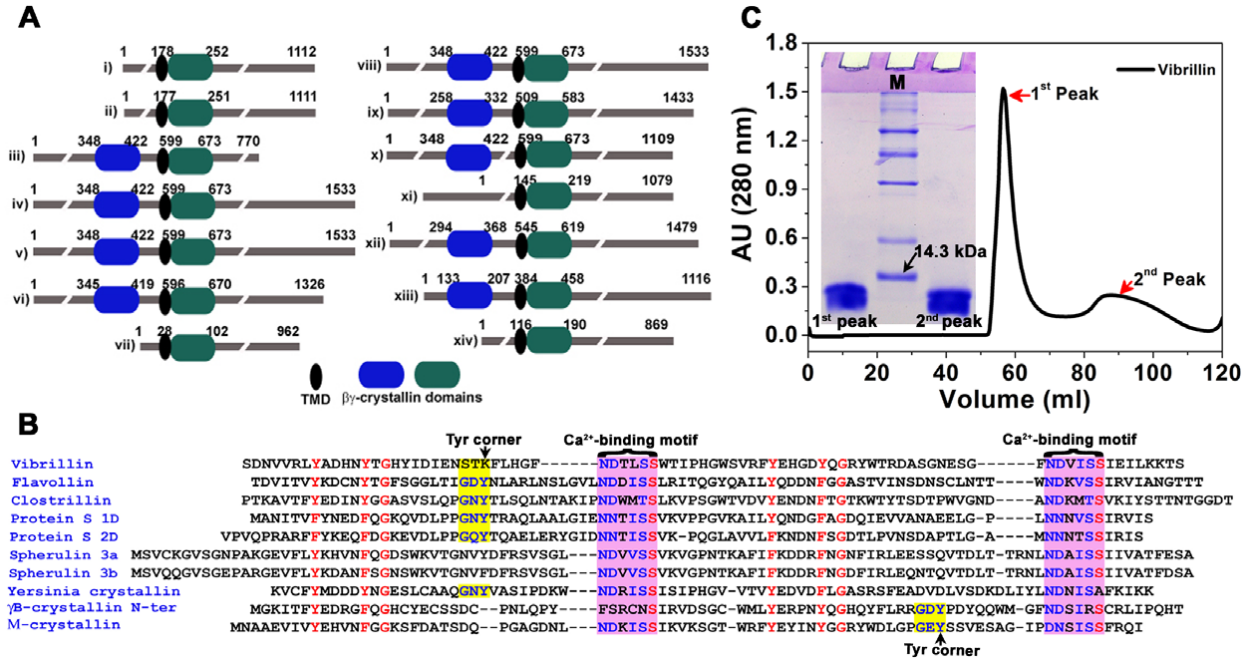
(**A**) βγ-Crystallin domain organization in the proteins (annotated as hypothetical or inner membrane proteins in data base) from different strains of *Vibrio* **(i)** putative inner membrane protein, [*Vibrio cholerae*] **NP_230470, (ii)** putative inner membrane protein [*Vibrio cholerae*] **AAK20779.1, (iii)** hypothetical protein VMD_00910 [*Vibrio mimicus* VM573] **ZP_05715045**, **(iv)** hypothetical protein VC274080_0898 [*Vibrio cholerae* 2740-80] **ZP_01675500, (v)** hypothetical protein VCV52_0783 [*Vibrio cholerae* V52] **ZP_01679275, (vi)** hypothetical protein VCV51_0554 [*Vibrio cholerae* V51] **ZP_04918684, (vii)** putative inner membrane protein [*Vibrio cholerae* O395] **YP_001216303 (viii)** hypothetical protein SX4_2180 [*Vibrio mimicus* SX-4] **EGU20577**, **(ix)** inner membrane protein [*Vibrio cholerae* HC-70A1] **EGS50069**, **(x)** hypothetical protein VCHC61A1_1747 [*Vibrio cholerae* HC-61A1] **EHI06219.1**, **(xi**) putative inner membrane protein, partial [*Vibrio cholerae* HC-48B2] **EHI08664.1**, **(xii**) inner membrane protein, partial [*Vibrio cholerae* HC-33A2**] EHI02422**, **(xiii)** inner membrane protein [*Vibrio cholerae* HC-40A1] **EGS51605.1 1** and **(xiv)** inner membrane domain protein [*Vibrio cholerae* HC-48A1] **EGS50981.1**. The sequence of the βγ-crystallin domain of above proteins is identical, observed at different positions marked in figure such as protein from *Vibrio cholerae* O395 strain is identical to hypothetical protein VC274080_0898 [*Vibrio cholerae* 2740-80] **ZP_01675500** from 572 to 1533 amino acids. The first βγ-crystallin domain (blue) is found only in proteins of some strains, while second βγ-crystallin domain (green) is common in all the members, thus selected for this study. (**B**) Sequence alignment of vibrillin domain with other βγ-crystallin domains. Sequences of Flavollin from *Flavobacterium johnsoniae*, Clostrillin from *Clostridium beijerncki*, Protein S (both domains) from *Myxococcus xanthus*, Spherulin 3a and 3b from *Physarum polycephalum*,Yersinia crystallin from *Yersinia pestis*, γ-crystallin from *Bos taurus* and M-crystallin from *Methanosarcina acetivorans* are aligned. Letters in red colour depicts the signature sequence of Greek key motifs. Ca^2+^-binding motifs -NDXXSS- are shown in the sequence. (**C**) Purification of vibrillin as octamer and dimer by gel filtration on a Superdex-75 column.

A hypothetical protein of 1533 amino acid residues (accession number ZP_01675500) in *Vibrio cholerae* 2740-80 genome was found to contain two putative βγ-crystallin domains (annotated at residues 348–422 and 599–673), predicted by the presence of the signature sequence (Figure 1A). The homologous protein (with varying sizes) is present in the genomes of twelve different strains of *Vibrio cholerae* and two strains of *Vibrio mimicus*, which are annotated as hypothetical proteins, except in two cases where they are named as putative inner membrane proteins probably because of the presence of a short transmembrane domain in the region of 572–594 amino acids (Figure 1). This transmembrane domain sequence is located before the second βγ-crystallin domain in all the strains. The full length *Vibrio* specific-protein is comprised of 1533 residues whereas the deposited sequences of proteins from other *Vibrio* strains are not of similar sequences length, though the sequences are identical (Figure 1). The second βγ-crystallin domain is common in all the sequences reported so far in the database for *Vibrio* strains. Vibrillin domain (84 amino acids) was cloned spanning the second domain with additional residues at both ends. This protein is not found in the genome sequence of any other organism available in the database.

The sequence of this domain does not have a Tyr corner (a signature sequence with GXY) and a Trp corner in B-type motif, though other characteristic signatures distinguishing a βγ-domain, such as YXXXXY/FXG and N/D-N/D-X-X-S/T-S sequences are present in both the Greek key motifs (Figure 1B). The latter sequence is indicative of the presence of two canonical Ca^2+^-binding motifs in the domain. Based on these atypical features, we selected this domain (84 amino acids, molecular mass 9.6 kDa) for further analysis.

While purifying we noticed that this protein elutes largely as two peaks during gel filtration chromatography (data discussed below), seen as a single band in SDS-PAGE. We, therefore, investigated the nature of association of this domain in solution.

### Homo-domain association

#### (i) Dynamic association/dissociation between octameric and dimeric forms

The purified vibrillin domain, when analysed for its molecular mass by size exclusion chromatography, was found to elute in two major peaks, suggesting that vibrillin exists largely in two populations in solution as re-confirmed by SDS-PAGE (Figure 1C) and spectral properties (discussed below). The major fraction of protein (1^st^ peak) was more symmetrical and eluted at >66 kDa equivalent to oligomer (octameric form) while a minor fraction (a broad 2^nd^ peak), eluted largely as a dimer (Figure 1C). It has been further noted that the concentration of protein subjected for gel filtration is a determinant for the relative proportions of both the species in a solution.

**Figure 2:**
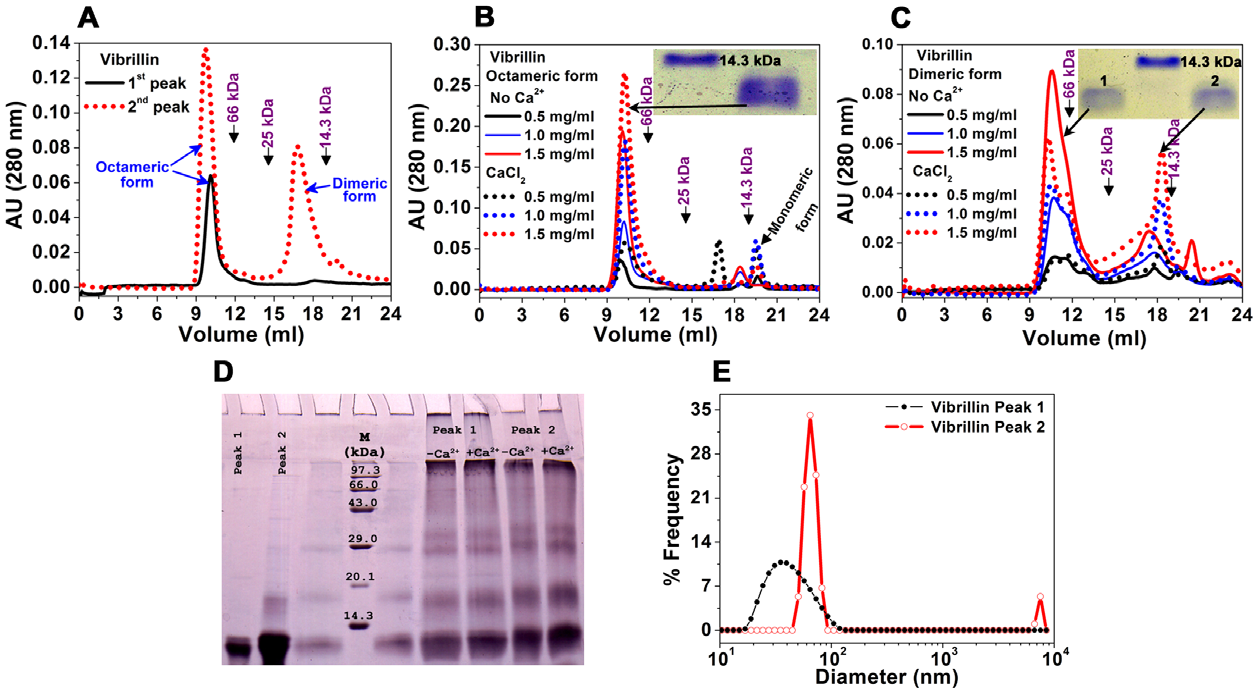
Octamer to dimer association of vibrillin domain. (**A**) Re-association of dimeric vibrillin to octamer and dimer in equilibrium. 500 µg protein both from octameric (peak 1) and dimeric (peak 2) fractions was subjected to gel filtration on Superdex-75 column, shown in solid black and dotted red line respectively. Concentration and Ca^2+^-dependent association of vibrillin: (**B**) peak 1, and (**C**) peak 2. The protein at three concentrations varying from 0.5 mg to 1.5 mg/ml was analysed on a Superdex-75 column in the presence of either EDTA (solid line) or CaCl_2_ (dotted line). Elution volumes of molecular mass standards are marked by arrows. (**D**) Glutaraldehyde crosslinking of both forms (octameric and dimeric) of vibrillin. Higher oligomers (tetramer, octamer) in both fractions are resolved on 15% SDS-PAGE. (**E**) Large oligomers formation of both peak 1 (oligomeric) and peak 2 (dimeric) vibrillin obtained by dynamic light scattering are shown by black (•) and red (○) respectively.

We next examined if there was any exchange between these two forms. When the oligomeric form (peak 1) was further analysed to size exclusion chromatography on the same column, it eluted largely in the same state (octameric state). On the contrary, the dimeric fraction (being minor) was concentrated before being re-subjected to the column, after which it eluted as two peaks, oligomers and dimers (Figure 2A). These results suggest that though the protein has greater propensity to form oligomers, this oligomerization is concentration-dependent. An additional peak corresponding to monomer at low concentrations of protein (<0.5 mg/ml), was noticed in gel filtration profile, suggesting that oligomers dissociate further if the protein concentration is reduced, and *vice versa* (Figure 2B).

A minor fraction of monomeric population seen at 1 and 1.5 mg/ml protein concentrations in the absence of CaCl_2_ disappears in the presence of Ca^2+^. In the presence of Ca^2+^, monomeric fraction is seen only when protein concentration is further reduced (0.5 mg/ml) (Figure 2B). On the other hand, the dimeric population of vibrillin appears to associate as well as dissociate to monomer, dimer, and oligomer (at concentrations between 0.5–1.5 mg/ml) in the absence of CaCl_2_. The oligomeric population elutes as a broad peak (octamer) with a small hump (likely hexamers as per molecular size). In the presence of Ca^2+^, vibrillin elutes only as dimers and oligomers. The oligomeric fraction elutes as a broad peak while monomeric fraction was absent in the concentrations used for analysis (0.5–1.5 mg/ml) (Figure 2C).

#### (ii) Formation of higher oligomers

**Figure 3:**
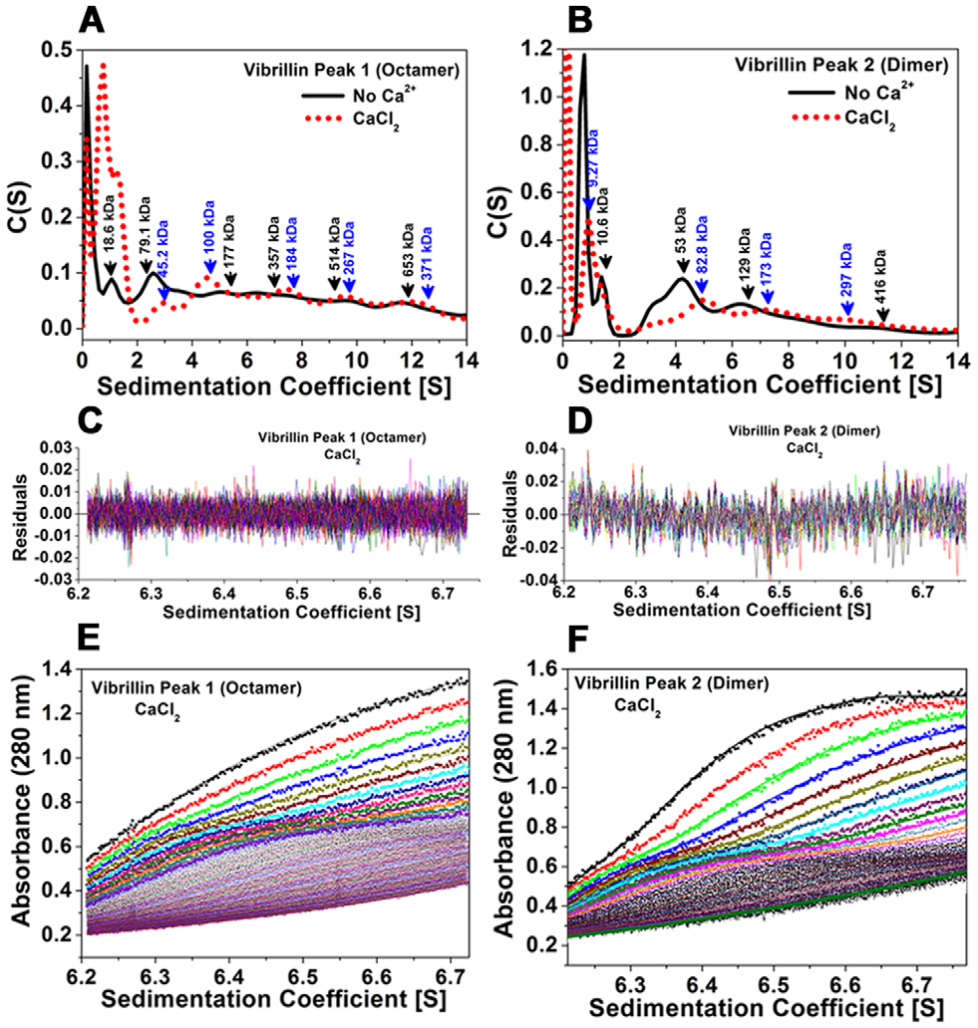
Sedimentation velocity profiles of vibrillin in the presence or absence of Ca^2+^. (**A, B**) Distribution of sedimentation coefficients of vibrillin (peak 1 and peak 2) in the presence or absence of Ca^2+^ shown in solid (black) and dotted (red) lines respectively. (**C, D**) Residuals of fitting and (**E, F**) movement of sedimenting boundary of vibrillin (peak 1 and 2) in the presence of Ca^2+^.

Vibrillin associates as oligomers, as demonstrated by glutaraldehyde crosslinking, dynamic light scattering and analytical ultra centrifugation. Both forms (dimeric as well as oligomeric) have a propensity to form dimer, tetramer as well as octamers as seen by glutaraldehyde crosslinking (Figure 2D). Very large oligomers could not penetrate the SDS-PAGE pores, though higher oligomers were clustered above 94 kDa marker and could not be resolved by SDS-PAGE. The dynamic light scattering of vibrillin (peak 1 and peak 2) also exhibits the polydisperse nature of the protein with more polydispersity of oligomeric fraction (molecular size range 20–100 nm) and a comparatively narrow range of dimeric fraction (80 nm) (Figure 2E). Oligomers were observed by analytical ultracentrifugation indicating the polydisperse nature of the protein (Figure 3). The major populations of 18.6 and 79 kDa representing the dimer and octamer were observed in sedimentation profile of peak 1 (oligomeric) vibrillin (Figure 3A, Figure S3). In addition, further higher oligomers were also seen, though they were not resolved by gel filtration as well as by glutaraldehyde crosslinking obviously due to low level and sensitivity of measurement. Based on analytical ultracentrifuge which is more precise technique, we cannot rule out the presence of higher oligomeric populations. The sedimentation velocity data obtained for vibrillin (peak 2 of Figure 1) exhibit the major populations of 10.6 and 53 kDa, which may represent the monomer and hexamer species; however, higher oligomers of 129 and 416 kDa are also seen as minor fraction (Figure 3B). Ca^2+^ helps in dissociating higher oligomeric (both oligomeric and dimeric) fractions to lower molecular mass populations as compared to its apo forms (Figure 3, Figure S3).

### Ca^2+^-binding properties and conformation

**Figure 4:**
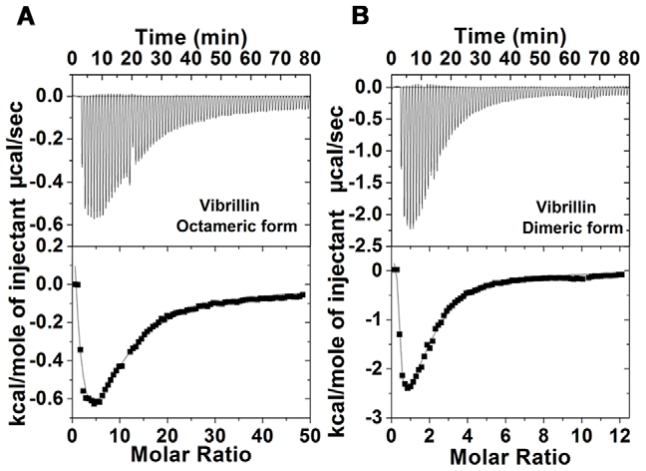
Ca^2+^-binding to octameric and dimeric vibrillin by ITC: (A) octameric and (B) dimeric fractions of vibrillin. The buffer used for preparation of ligand and protein for ITC experiment was 50 mM Tris-Cl (pH 7.0), 100 mM KCl. The concentrations of protein and Ca^2+^ used in ITC experiments were 100 µM and 10 mM respectively. The protein in a sample cell was titrated with Ca^2+^ which was loaded in a syringe with 80 injections of 3 µl each from a stock of 10 mM CaCl_2_.

As vibrillin domain has two canonical Ca^2+^-binding motifs, i.e., NDTLSS and NDVISS, in each Greek key motif (Figure 1B), Ca^2+^-binding to: (i) octameric, and (ii) dimeric fraction was undertaken by ITC. Ca^2+^-binding to vibrillin followed exothermic heat changes as seen in thermograms; (Figure 4A, B). However, due to polydisperse nature of the protein, it is not appropriate to calculate the exact binding stoichiometry. Mg^2+^ does not bind to vibrillin, suggesting specificity for Ca^2+^ (Figure S4).

**Figure 5:**
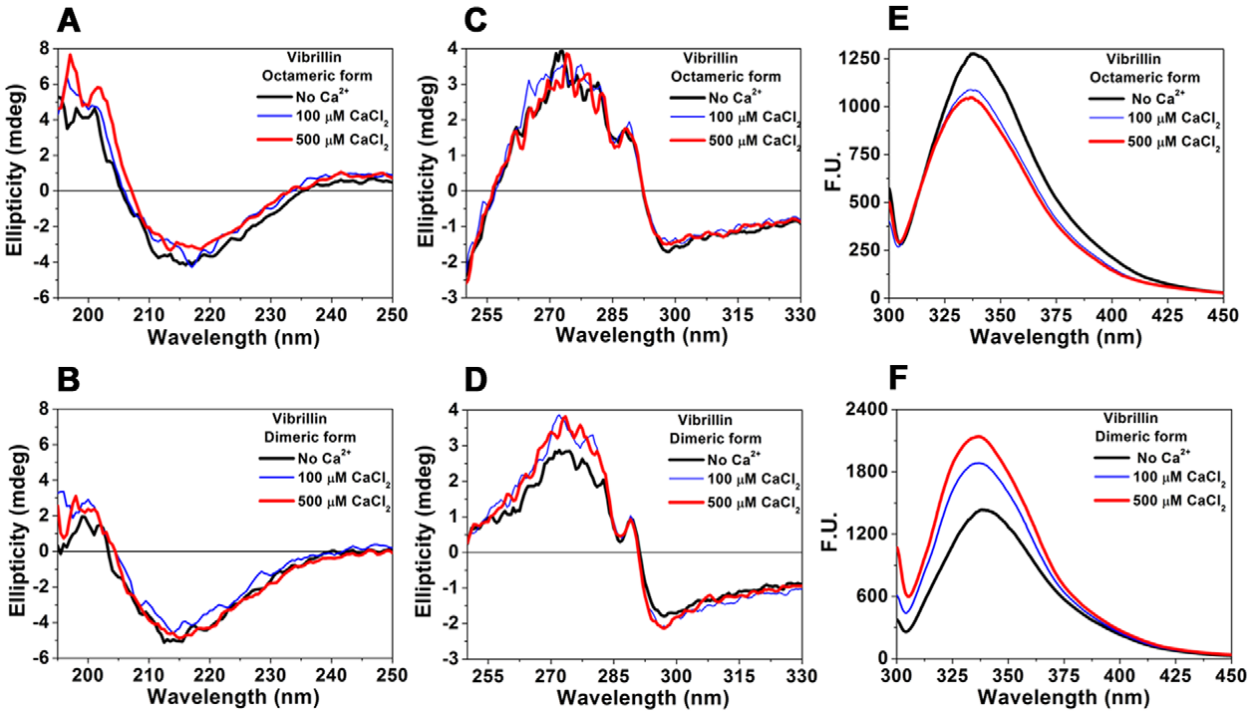
Ca^2+^-binding conformational changes monitored by Far-UV and near-UV CD spectra: (A, C) octameric, and (B, D) dimer forms of vibrillin. The protein concentrations for far- and near-UV CD measurements were 250 µg/ml and 1 mg/ml respectively. CaCl_2_ was added to final concentrations of 0, 0.1 and 0.5 mM. No significant changes were observed in near-UV CD of both forms of vibrillin upon Ca^2+^ titration (10 µM–5 mM of CaCl_2_). **(E, F)** Change in intrinsic Trp fluorescence of vibrillin (octameric and dimeric forms) (0.1 mg/ml) upon binding Ca^2+^ (0, 0.1 and 0.5 mM of CaCl_2_).

Both forms of vibrillin domain exist predominantly in β-sheet conformation with a minimum at 215 nm in far-UV CD. We observed insignificant change in far-UV CD spectra upon Ca^2+^-binding (Figure 5A, B). Both fractions have well defined, tertiary fold as seen in near-UV CD as (Figure 5C, D). Upon Ca^2+^-binding, significant conformational changes were seen in Trp fluorescence. The octameric form showed λ_max_ at 338 nm and fluorescence intensity quenched upon Ca^2+^-binding with a blue shift in λ_max_ to 336 nm (Figure 5E). The λ_max_ of the dimeric form of apo vibrillin protein was 342 nm while it was shifted to 338 nm (4 nm blue shift) in the Ca^2+^-bound form (Figure 5F). It is inferred from the fluorescence data that the local micro-environment surrounding Trp residues is perturbed with either exclusion of polar solvent from hydrophobic regions or movement of side chain hydrophobic residue to more non-polar environments.

### Oligomerization increases thermal stability

**Figure 6:**
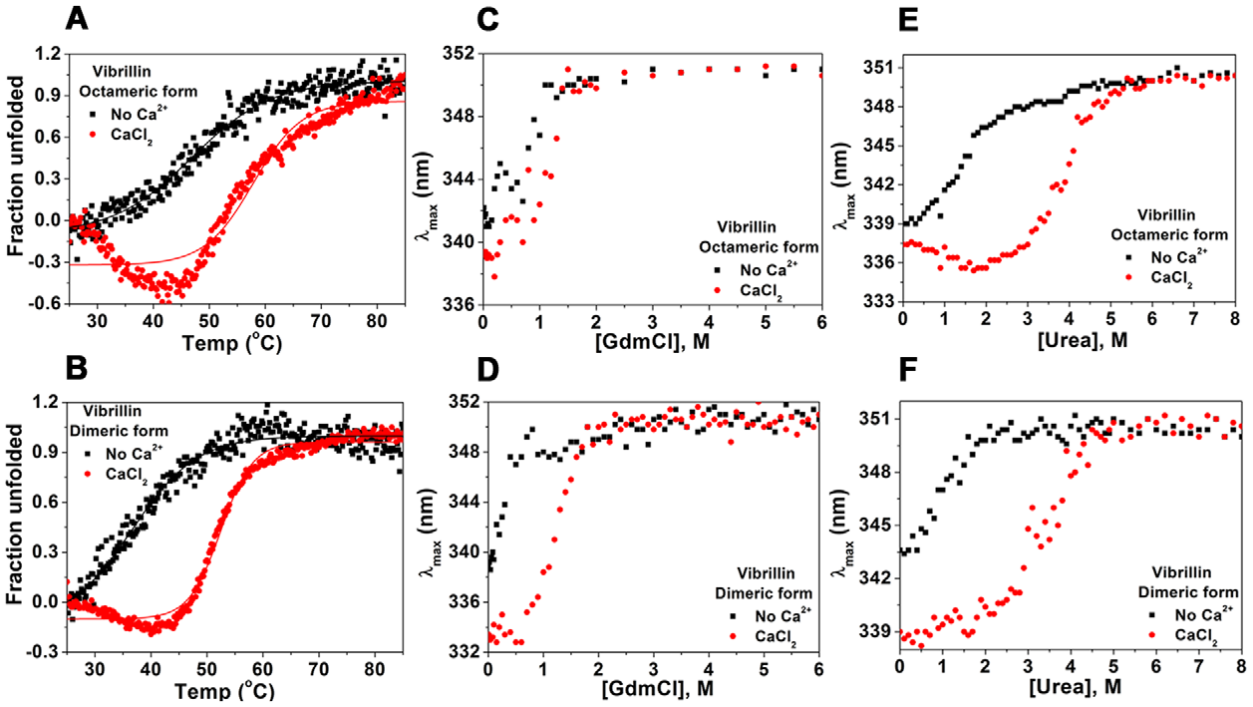
Differential stabilization of octameric and dimeric vibrillin by Ca^2+^: (A) octameric form, and (B) dimeric form, demonstrating significant increase in T_m_ (∼10°C in octamer and 14°C in dimer forms) in the presence of CaCl_2_. GdmCl- and urea-induced equilibrium unfolding of vibrillin (both octamer and dimer) in the absence or presence of CaCl_2_. Changes in wavelength emission maxima (λ_max_) of vibrillin: **(C)** octameric, and **(D)** dimeric with [GdmCl]. Changes in λ_max_ of vibrillin: **(E)** octameric and **(F)** dimeric with [urea].

Apo oligomeric vibrillin domain is thermally more stable (T_m_ 46.9°C) than the apo dimeric form (T_m_ 36.5°C) (Figure 6). The thermal unfolding profiles in both the cases are different, apo-dimeric form follows a more cooperative transition than the apo oligomeric form. Ca^2+^-binding increases the thermal stability in both the cases significantly (ΔT_m_∼10–14°C), but oligomeric form of vibrillin is more stable than its dynamic dimeric form (Figure 6A, B). In the presence of Ca^2+^, a significant change in ellipticity in 25–45°C prior to transition zone was also noticed during thermal unfolding of oligomeric vibrillin (peak 1), which was less pronounced in case of dimeric mixture (peak 2).

### Equilibrium unfolding of vibrillin: different association form, different unfolding transitions

Equilibrium unfolding of oligomeric form was monitored by Trp fluorescence in the absence or presence of Ca^2+^. Fluorescence intensity increased in samples up to 0.7 M GdmCl followed by a gradual decrease with no change in emission maxima. Beyond 0.7 M GdmCl, there was a sharp red shift in emission maximum with complete exposure of Trp residues (λ_max_ 352 nm) at 1.5 M GdmCl (Figure 6C, Table 1). Only a gradual increase in fluorescence intensity was seen in samples containing 0.7 to 6 M GdmCl concentration.

**Table 1:** Fluorescence-derived thermodynamic parameters obtained from unfolding transitions of both forms (octameric and dimeric) of vibrillin.

Vibrillin (Octameric and dimeric form)	aΔG^0^ kcal mol^−1^		bm kcal mol^−1^M^−1^		^c^c_1/2_ [M]	
	EDTA	CaCl_2_	EDTA	CaCl_2_	EDTA	CaCl_2_
**Dimeric Vibrillin (GdmCl)**	1.4±0.14	3.1±0.17	1.6±0.08	2.2±0.11	0.9	1.4
**Octameric Vibrillin (GdmCl)**	*nf		-		-	
**Dimeric Vibrillin (Urea)**	1.1±0.08	3.3±0.18	0.8±0.05	0.9±0.04	1.3	3.9
**Octameric Vibrillin (Urea)**	1.4±0.08	5.1±0.21	0.7±0.04	1.2±0.05	1.9	4.1

* n.f.: non-fittable,

a Free energy of unfolding,

b Cooperativity index of the transition and ^c^Calculated denaturant concentration at transition midpoint.

The wavelength emission maxima (λ_max_) of dimeric vibrillin was 338 nm in the absence of Ca^2+^ while a 6 nm blue shift (λ_max_ 332 nm) was observed in the presence of CaCl_2_. Dimeric vibrillin follows red shift in wavelength maximum gradually up to 0.8 M GdmCl with complete exposure of Trp residues (λ_max_ 352 nm) at 1.5 M GdmCl in the absence of Ca^2+^. The unfolding data of apo dimeric vibrillin could not fit properly, however, in the presence of Ca^2+^, it follows a two-state transition (Figure 6, Table 1). A significant gain in stability was observed in the presence of Ca^2+^ for both fractions during urea-induced unfolding (Figure 6E, F).

**Figure 7:**
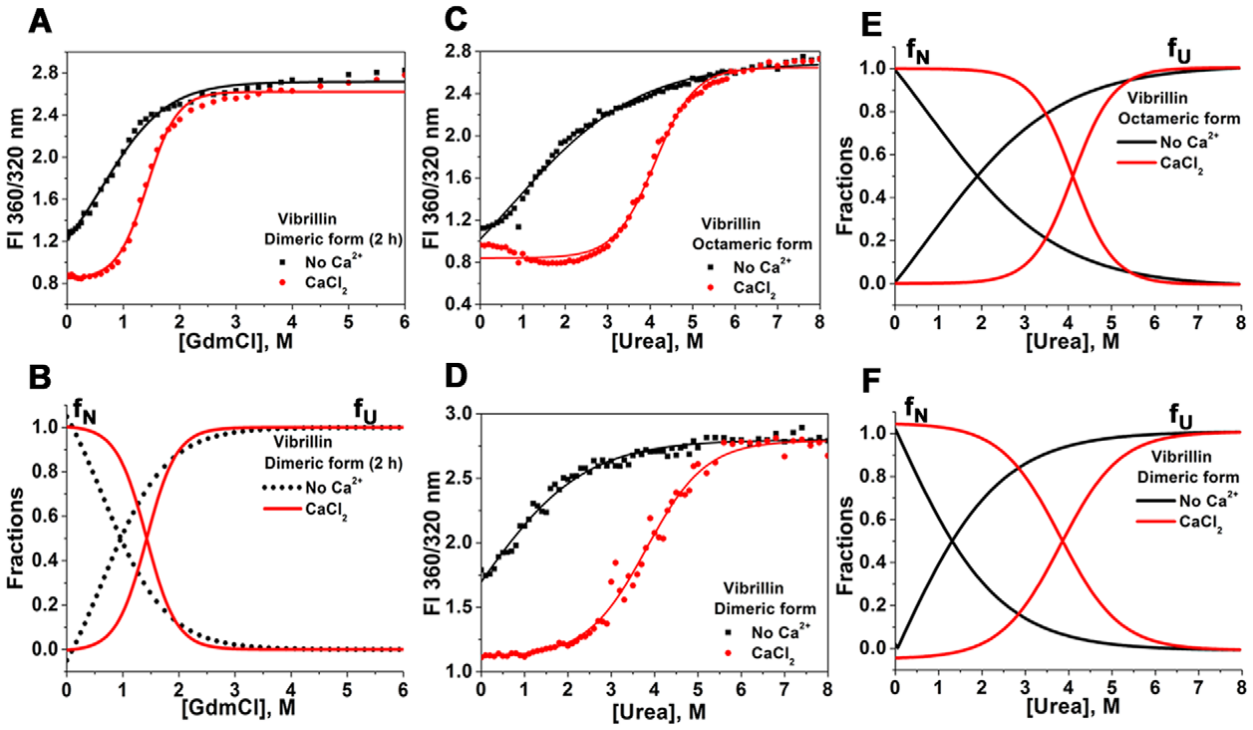
GdmCl-induced equilibrium unfolding of dimeric vibrillin in the absence or presence of CaCl_2_. **(A)** Changes in ratios of fluorescence intensity at wavelengths (360 nm and 320 nm) and **(B)** best fit in the two-state unfolding process with [GdmCl]. The significant change in the c_1/2_ value was observed in the holo states (1.4 M) of vibrillin as compared to apo protein (0.9 M). Urea-induced equilibrium unfolding of vibrillin (both forms) in the absence or presence of CaCl_2_. Changes in ratios of fluorescence intensity of **(C)** octameric, and **(D)** dimeric vibrillin at wavelengths (360 nm and 320 nm), and **(E, F)** best fits to two-state unfolding process of both forms of vibrillin with urea. f_N_ and f_U_ represents the fractions at native and unfolded states respectively. The significant change in c_1/2_ value was observed in the holo state (4.1 M) of octameric vibrillin as compared to apo protein (1.9 M). Dimeric vibrillin also shows significant increase in c_1/2_ value (3.9 M for holo as compared to 1.3 M apo) upon Ca^2+^-binding.

While GdmCl-induced unfolding data of oligomeric form could not fit to any model and Ca^2+^ apparently does not influence stability, Ca^2+^ increases the stability of the dimeric fractions (c_1/2_ apo and holo proteins are 0.9 and 1.4 M, ΔG 1.4 and 3.1 kcal mol^−1^ respectively) (Figure 7A, B Table 1). Urea-induced unfolding of both fractions indicated a two-state unfolding in the absence or presence of Ca^2+^, plotted by the ratio of fluorescence intensity at 360/320 nm (Figure 7C–F). As seen, Ca^2+^ increases the stability of the dimer significantly (c_1/2_ apo and holo proteins are 1.3 and 3.9 M). A similar gain in stability was observed for oligomeric fraction of vibrillin (c_1/2_ apo and holo proteins are 1.9 M and 4.1 M) (Figure 7). In the presence of Ca^2+^, ΔG observed for dimer and oligomeric fractions of vibrillin were 3.3 kcal mol^−1^ and 5.1 kcal mol^−1^, while in the absence of Ca^2+^, a significant decrease in ΔG was observed for both dimeric and oligomeric forms (1.1 and 1.4 kcal mol^−1^ respectively) of vibrillin (Table 1).

**Figure 8:**
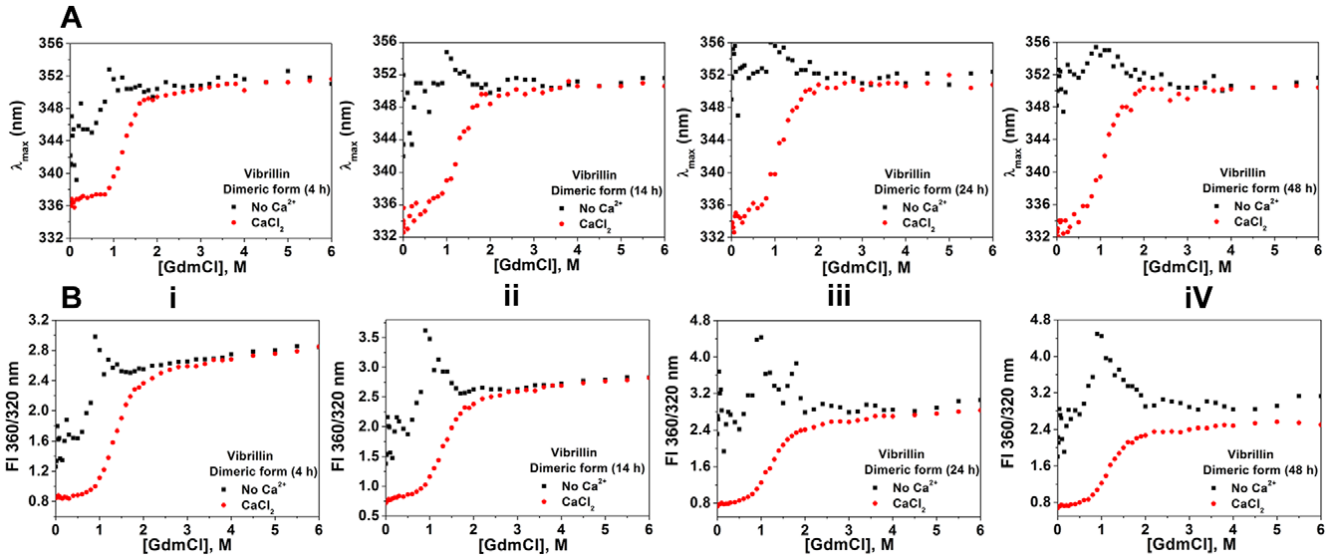
Equilibrium unfolding transitions of dimeric vibrillin with time. **(A)** Comparison of changes in λ_max_, and **(B)** Changes in ratios of fluorescence intensity at fixed wavelengths (360 nm and 320 nm) versus increasing concentrations of denaturant (GdmCl) of vibrillin at **(i)** 4 hours, **(ii)** 14 hours, **(iii)** 24 hours and **(iv)** 48 hours of incubation with [GdmCl]. The data obtained in the presence of CaCl_2_ was fit to a two-state equation. The data obtained in the absence of CaCl_2_ could not fit to any equation, due to non-linearity from 0.5 to 1.5 M GdmCl concentrations.

While there was no apparent change in unfolding transitions of holo protein with time (measured as ratio of fluorescence intensity at 360/320 nm at 2, 14, 24 and 48 hours), apo protein unfolding transitions were not linear (scattered data) up to 1.8 M GdmCl concentration (Figure 8). The non-linearity in fluorescence intensity of apo vibrillin domain after 4 h of incubation may be due to invisible aggregation of partially unfolded species (up to 1.8 M GdmCl), which is inhibited in the presence of Ca^2+^.

#### (i) Unfolding intermediates of oligomeric and dimeric vibrillin

**Figure 9:**
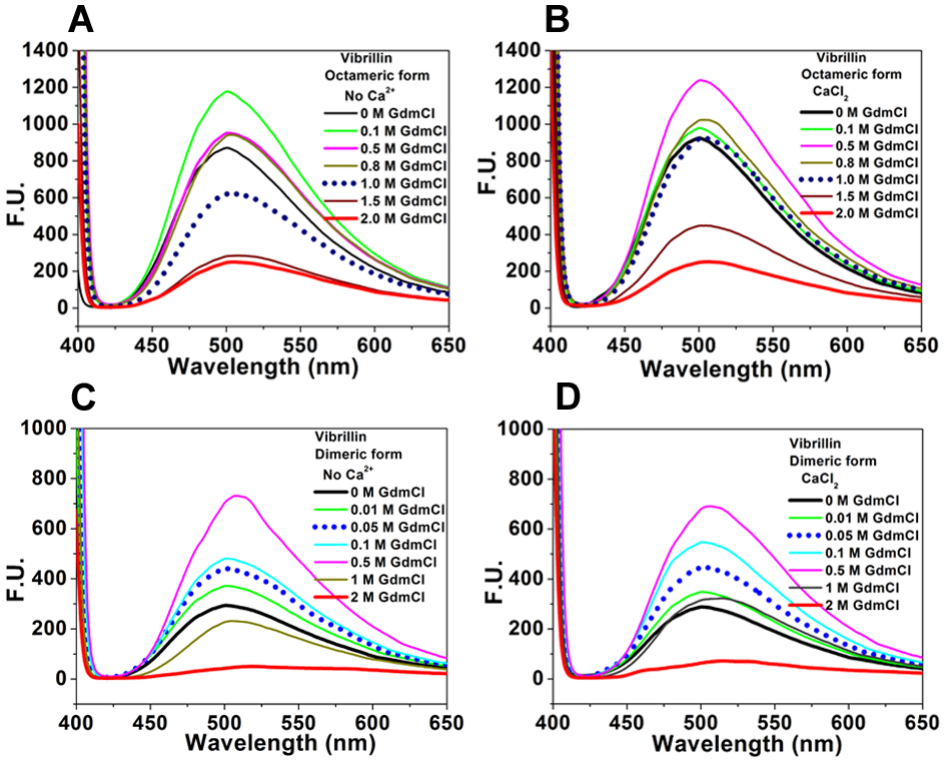
Intermediate state of unfolding of vibrillin identified by measuring surface hydrophobicity. **(A, B)** octameric, and **(C, D)** dimeric forms in GdmCl monitored by bis-ANS fluorescence in the absence or presence of CaCl_2_ (5 mM) and increasing concentrations of GdmCl. bis-ANS (10 µM) was added to the protein solution and incubated for 30 min; increased fluorescence of bis ANS–protein complex (dimeric form) both in the absence or presence of Ca^2+^ (up to 0.5 M GdmCl) demonstrating the presence of stable intermediate states.

The unfolding of vibrillin domain follows two-state transition, when data are analyzed by fitting in the equation described in methods. However, we could identify an intermediate state of unfolding of both forms of vibrillin in the sub-molar (non-denaturing) concentrations of GdmCl by monitoring the change in surface hydrophobicity using bis-ANS fluorescence. A sharp increase in surface hydrophobicity (bis-ANS fluorescence) of oligomeric and dimeric vibrillin both in the absence or presence of Ca^2+^ was observed in protein samples in 10 µM to 500 mM GdmCl, which decreased at higher denaturant concentrations (Figure 9). These results suggest the presence of an on-pathway unfolding intermediate (UI) state at sub-molar concentrations of denaturant [16]. This is not similar to that seen in lens γ-crystallin, where only partially refolded but not unfolded species form insoluble aggregates [17]. Interesting differences were noted in the intermediate states of both forms. While UI state of octameric apo-vibrillin is formed at 0.1 M GdmCl, Ca^2+^ shifts UI state towards higher GdmCl concentration (0.5 M) (Figure 9A, B). Incidentally, there is no such shift in UI state of dimeric vibrillin by Ca^2+^ which is formed at 0.5 M GdmCl concentration both in the apo and holo forms (Figure 9C, D). Increased fluorescence intensity at resonance wavelength (395 nm) was observed in samples in 0.2–0.5 M concentrations of GdmCl, both in the absence or presence of CaCl_2_ suggesting a possibility of soluble aggregation of intermediate states (Figure S1).

#### (ii) Unfolding intermediates of oligomeric and dimeric vibrillin have different quaternary association

**Figure 10:**
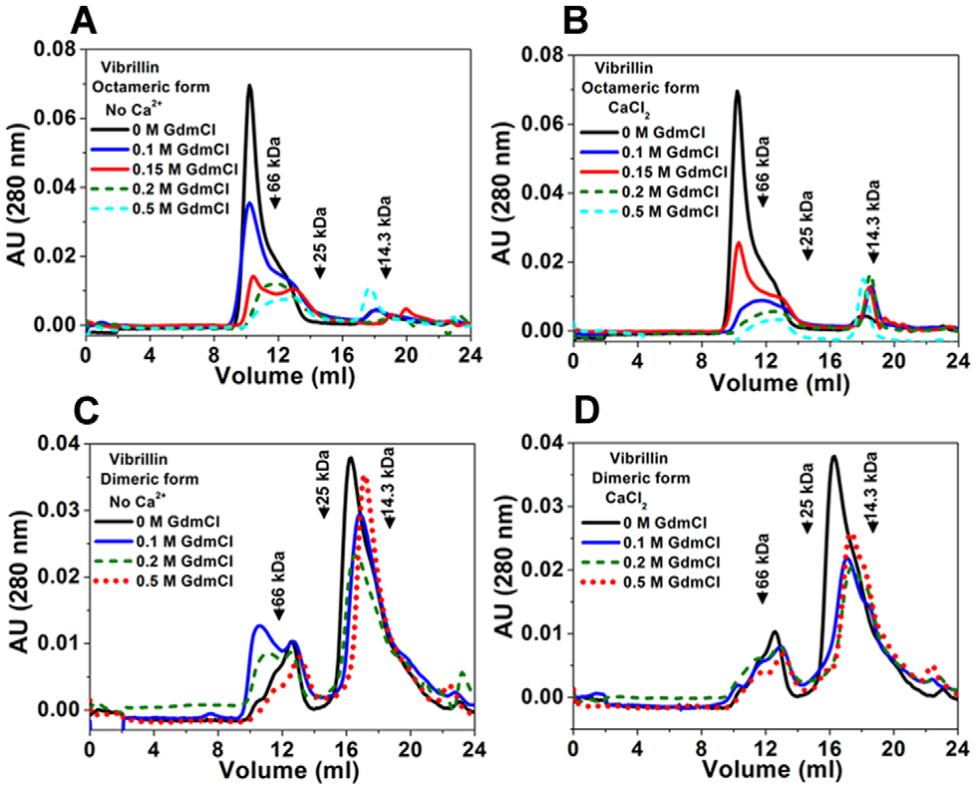
Quaternary association of unfolding intermediates of octamer and dimer vibrillin. **(A)** octameric vibrillin does not dissociate and re-elutes as octamer as a major fraction in gel filtration in the absence of CaCl_2_. **(B)** In the presence of CaCl_2_, it elutes as octamer and dimer (minor fraction) with increasing concentrations of GdmCl. **(C)** and **(D)** Association of dimeric fraction of vibrillin to larger oligomer (hexamer, octamer) and dimer at 0.1 M–0.5 GdmCl both in the absence or presence of CaCl_2_. 0.3 mg/ml of vibrillin (collected separately from octameric and dimeric forms) were re-loaded on a Superdex-75 column, in the presence of either EDTA or CaCl_2_ in concentrations varying from 0.1 to 0.5 M GdmCl. Elution volumes of molecular mass standards are marked by arrows. The reduction in absorbance was observed in the presence of GdmCl in octameric form due to precipitation while no such precipitations were observed for dimeric form. The elution was not corrected for the change in the viscosity of buffer.

We next compared the quaternary association of UI states using gel filtration. UI state of apo-oligomeric vibrillin (at 0.1 M GdmCl) has octameric (a peak hump as hexamer) association with only a minor fraction as dimer (Figure 10 A). At 0.5 M GdmCl in the presence of Ca^2+^, UI state of holo-oligomeric vibrillin has almost similar gel filtration chromatogram (Figure 10 B). The comparative less peak height indicates for protein precipitation, which we observed during unfolding due to aggregation, akin to that seen in the case of cataract associated mutants of γD-crystallin [18]. At 0.2 M GdmCl, Ca^2+^ influences the formation of dimer without any additional oligomer formation (Figure S2). Even at further GdmCl concentrations (0. 5 M), similar dissociation is seen, thus suggesting that while protein is partially unfolded, its oligomeric form is not dissociated completely.

Interestingly, UI state of dimeric vibrillin at 0.5 M GdmCl was observed in both apo and holo forms, as noticed by increased intensity of bis-ANS fluorescence (Figure 10 C, D). Furthermore, gel filtration profile also suggests that UI states of dimeric forms had propensity to form polydisperse species (octamer and hexamer) eluted as a peak with a hump. Ca^2+^-binding had no effects on UI state of dimeric form.

## Discussion

The *Vibrio* species specific protein containing βγ-crystallin domains has several atypical sequence features, some of them are noted in Greek key motifs of other members, such as in Yersinia crystallins [12]. Vibrillin domain shows properties not reported by any other βγ-crystallin domains. It forms concentration-dependent oligomers and exists in solution in several forms ranging from high oligomers to dimer (also monomer as a minor fraction if protein concentration is <0.5 mg/ml). It appears to be the only known βγ-domain which forms such oligomers, though many individual βγ domains form homodimer via domain pairing [19]. Individual domains of lens γB-crystallin do not associate and remain monomer in solution [20]. Ca^2+^ is not known to influence the domain association of other βγ-crystallins, including those with canonical Ca^2+^-binding motifs. On the other hand, Ca^2+^ shifts the association towards dimeric form at the loss of monomers at low protein concentrations (in the apo form) of vibrillin.

Vibrillin has two canonical Ca^2+^-binding motifs, i.e., NDTLSS and NDVISS, and binds Ca^2+^ as seen by ITC. The difference in binding affinities of βγ-crystallin domains is due to the variations in the amino acids in the N/D-/N/D-X1-X2-T/S-S motif [21]. Ca^2+^-binding affinities of various βγ domains range from 4 µM to 100 µM [22], [23]. The affinity for Ca^2+^ could not be calculated owing to polydisperse nature of vibrillin domain. This protein does not undergo any notable conformational changes, which is a common feature of most βγ-crystallins except those which are intrinsically unstructured in apo form [12], [13], [19], [24].

Since vibrillin sequence is not similar to lens βγ-crystallins, it serves as an interesting domain for comparing such distantly related proteins. It has been shown that stability of βγ-crystallin domains is tuned by N/D-/N/D-X1-X2-T/S-S motif [25]. Unfolding of vibrillin depicts several unusual facets, despite having two Ca^2+^-binding canonical motifs. This domain is not highly stable as seen by unfolding studies. While Ca^2+^ increases the stability of vibrillin protein to some extent when unfolded by GdmCl, the Ca^2+^-induced gain in stability is much more significant when unfolded by urea. An unfolded intermediate with more exposed hydrophobicity was also observed. Our data demonstrate novel facets of unfolding of an oligomeric protein. The intermediate state retains the original association (octamer) and does not dissociate to monomer, though Ca^2+^ favours more dissociation towards dimerization. On the other hand, the intermediate state of dimeric protein aggregates rather than dissociates. Partially unfolded intermediate species of oligomeric vibrillin tend to form insoluble aggregates, which is not similar to that seen in case of human γD-crystallin (which forms only during refolding) [18]. Several mutations in lens γ-crystallins are known to alter the properties making them more prone to aggregate in partially unfolded form [18], [26], [27]. Though molecular basis of such aggregation propensity is not known, it has been recently suggested that aggregation of γ-crystallin during refolding is due to C-terminal domain swapping [28]. While lens γ-crystallin may be considered as an extensively modified form of vibrillin domain (sequence similarities of 43% to N-terminal domain, and 36% to C-terminal domain), such studies could provide further clues for the non-crystallin like properties attained by these members with extensive sequence modifications.

A few microbial βγ-crystallins, such as Protein S from *Myxococcus xanthus* and Spherulin 3a are associated with stress in Ca^2+^-dependent form. As seen from our data on the role of Ca^2+^-mediated stabilization, vibrillin may not be involved in such stress-related function in *Vibrio*. It would be important to investigate its role in biofilm formation as its formation is known to play a role in *Vibrio* pathophysiology and survival strategies [29]. Incidentally, Ca^2+^ has been implicated in decreasing the proteins related biofilm formation in *Vibrio* species [30], [31]. In summary, we have characterized a βγ domain of a *Vibrio*-specific protein. This domain associates in solution forming oligomers with a fraction of the population being in dimeric state. Ca^2+^-binding influences the self-association to some extent. Both oligomeric as well as dimeric populations form intermediates during unfolding which follow the similar pathways and dissimilar aggregation. It would be worth to explore the role of this protein in virulency or in *Vibrio*-specific functions.

## Supporting Information

Figure S1 **Change in the surface hydrophobicity (demonstrating the resonance peak) of (A, B) octameric and (C, D) dimeric vibrillin in the presence of GdmCl monitored by bis-ANS fluorescence.** Vibrillin samples were prepared in the absence or presence of CaCl_2_ (5 mM) at different concentrations of denaturant, GdmCl. bis-ANS (10 µM) was added to the protein solution and incubated for 30 min and samples were excited at 395 nm, emission spectra were recorded between 380 nm and 650 nm. The increase in scattering at 395 nm is seen at 0.2 to 0.5 M GdmCl in bis ANS-protein complex.
(TIF)

Figure S2 **Comparison of unfolding intermediates of vibrillin domain: (A) Octameric vibrillin eluted as octamer/hexamer in gel filtration Superdex-75 column in the absence of CaCl_2_.** In the presence of CaCl_2_, it eluted as dimer although octamer was also present at 0.2 M GdmCl. **(B)** Comparison of octameric and dimeric vibrillin in the absence of CaCl_2_ at 0.2 M GdmCl concentration. Ca^2+^ does not influence the elution profile of dimeric vibrillin at this concentration. 0.5 mg/ml of vibrillin (both octameric and dimeric) samples were subjected to a gel filtration Superdex-75 analytical column, in the presence of either EDTA or CaCl_2_ with 0.2 M GdmCl. Elution volumes of molecular mass standards are marked by arrows.
(TIF)

Figure S3 **Sedimentation velocity profile of vibrillin in the absence Ca^2+^.** **(A, B)** Movement of sedimenting boundary of both oligomeric (peak 1) and dimeric (peak 2) vibrillin. **(C, D)** Residuals of fitting for oligomeric and dimeric proteins in the absence of Ca^2+^.
(TIF)

Figure S4 **Mg^2+^-binding to vibrillin by ITC: The buffer used for preparation of ligand and protein for ITC experiment was 50 mM Tris-Cl (pH 7.0), 100 mM KCl.** The ITC experiment was performed under the similar conditions as for Ca^2+^-binding. Concentrations of protein and Mg^2+^ used in ITC experiments were 100 µM and 10 mM respectively. The protein in a sample cell was titrated with 80 injections of 3 µl each from a stock of 10 mM MgCl_2_.
(TIF)
