# Supplementary figures and images for: Association Properties and Unfolding of a βγ-Crystallin Domain of a Vibrio-Specific Protein
Source: PLoS One. 2013 Jan 22;8(1):e53610. doi: 10.1371/journal.pone.0053610 (PMC3551895; doi:10.1371/journal.pone.0053610)

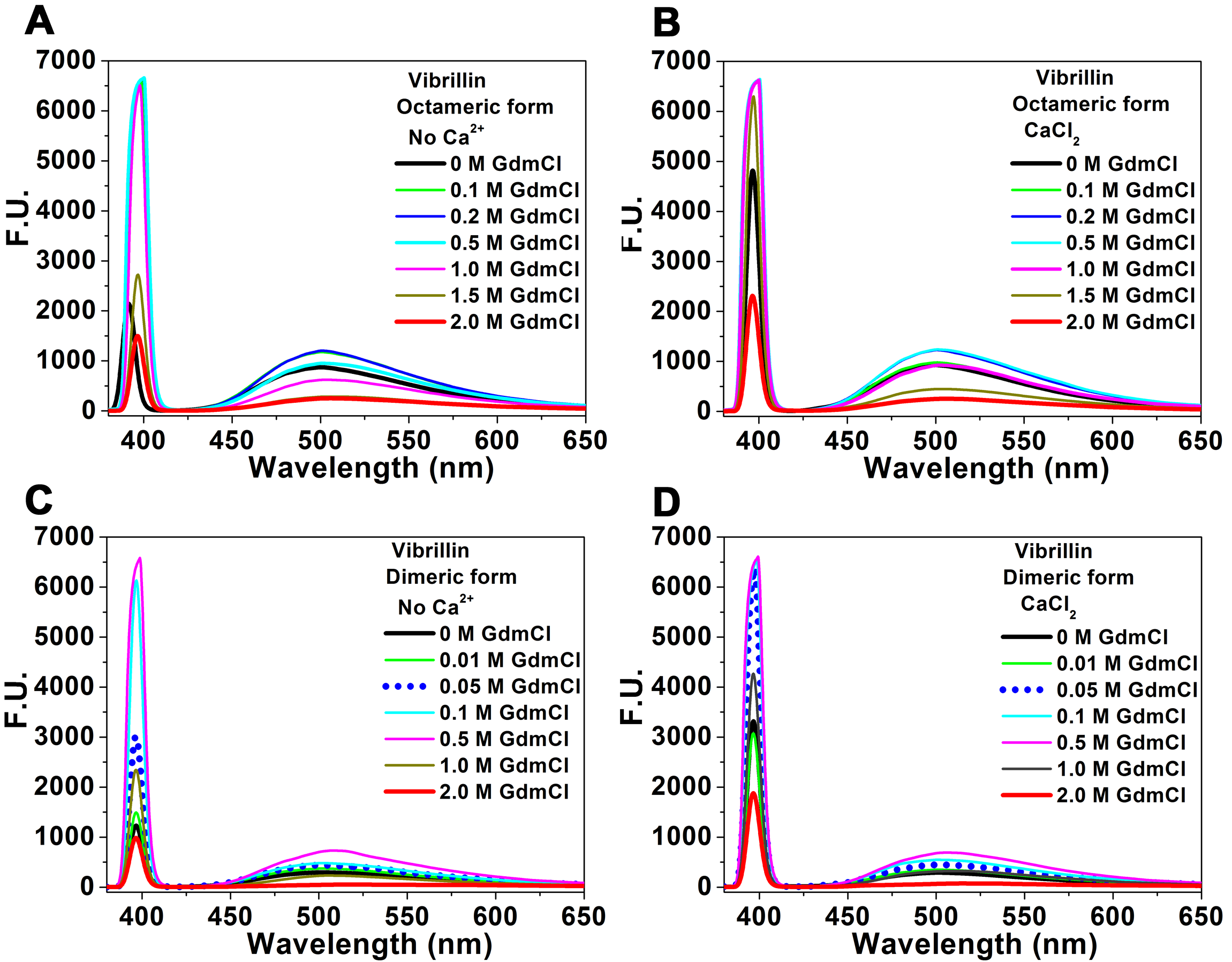

Supplement: Figure S1 — Change in the surface hydrophobicity (demonstrating the resonance peak) of (A, B) octameric and (C, D) dimeric vibrillin in the presence of GdmCl monitored by bis-ANS fluorescence. Vibrillin samples were prepared in the absence or presence of CaCl2 (5 mM) at different concentrations of denaturant, GdmCl. bis-ANS (10 µM) was added to the protein solution and incubated for 30 min and samples were excited at 395 nm, emission spectra were recorded between 380 nm and 650 nm. The increase in scattering at 395 nm is seen at 0.2 to 0.5 M GdmCl in bis ANS-protein complex. (TIF) [file pone.0053610.s001.tif]

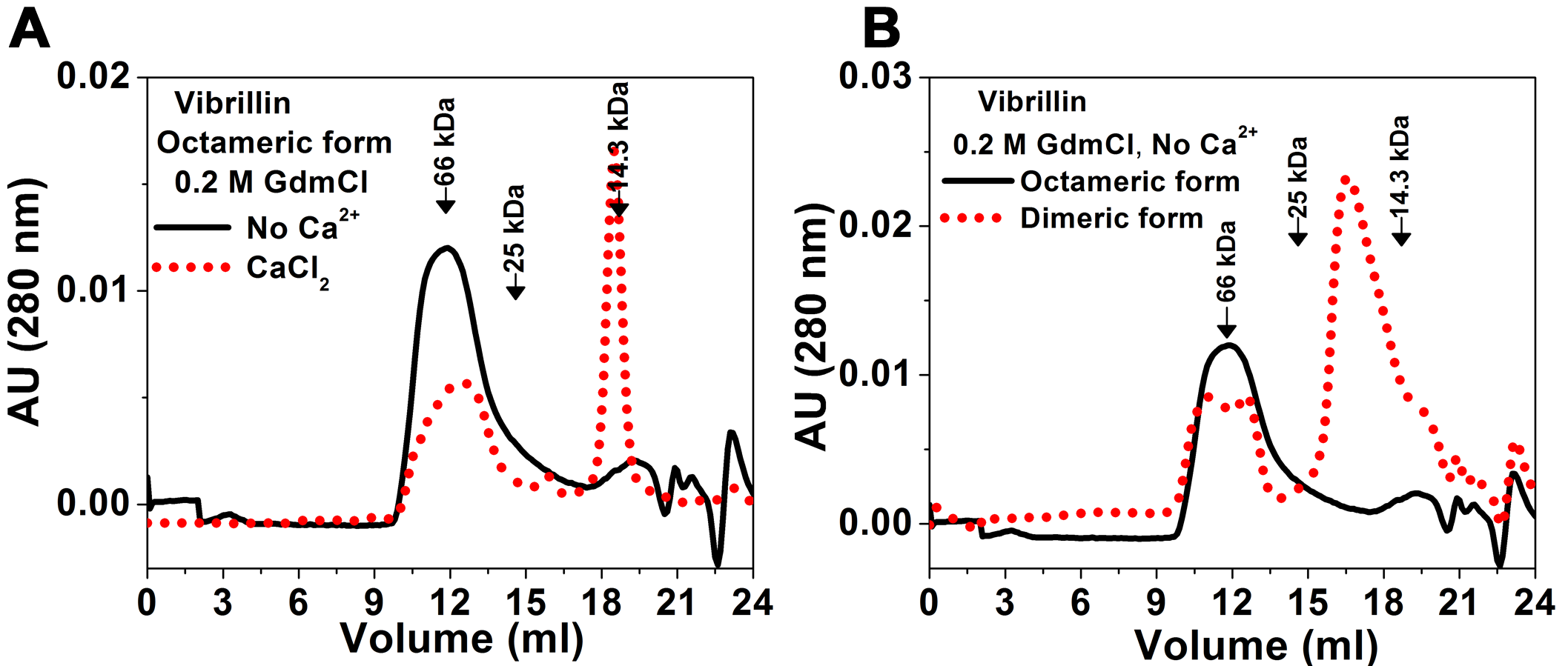

Supplement: Figure S2 — Comparison of unfolding intermediates of vibrillin domain: (A) Octameric vibrillin eluted as octamer/hexamer in gel filtration Superdex-75 column in the absence of CaCl2. In the presence of CaCl2, it eluted as dimer although octamer was also present at 0.2 M GdmCl. (B) Comparison of octameric and dimeric vibrillin in the absence of CaCl2 at 0.2 M GdmCl concentration. Ca2+ does not influence the elution profile of dimeric vibrillin at this concentration. 0.5 mg/ml of vibrillin (both octameric and dimeric) samples were subjected to a gel filtration Superdex-75 analytical column, in the presence of either EDTA or CaCl2 with 0.2 M GdmCl. Elution volumes of molecular mass standards are marked by arrows. (TIF) [file pone.0053610.s002.tif]

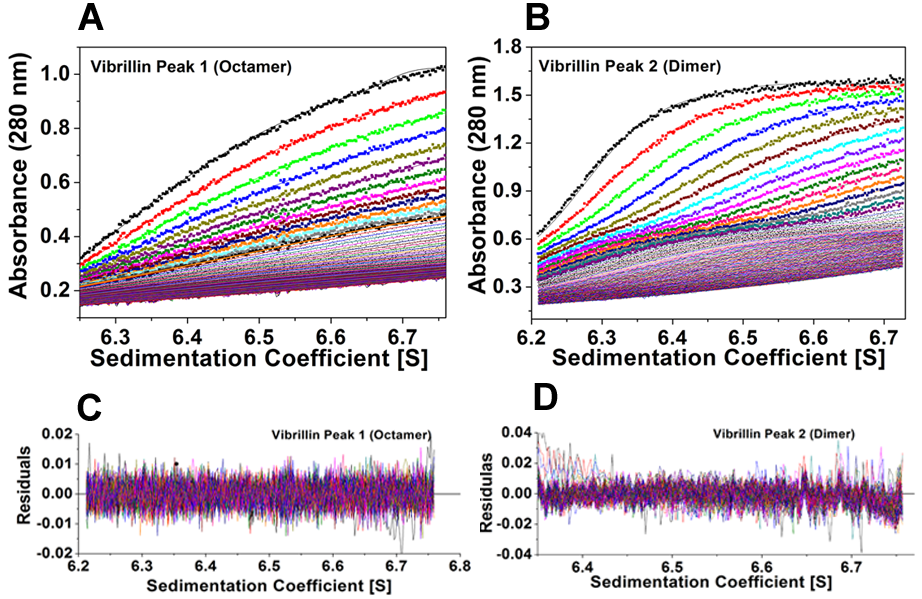

Supplement: Figure S3 — Sedimentation velocity profile of vibrillin in the absence Ca2+. (A, B) Movement of sedimenting boundary of both oligomeric (peak 1) and dimeric (peak 2) vibrillin. (C, D) Residuals of fitting for oligomeric and dimeric proteins in the absence of Ca2+. (TIF) [file pone.0053610.s003.tif]

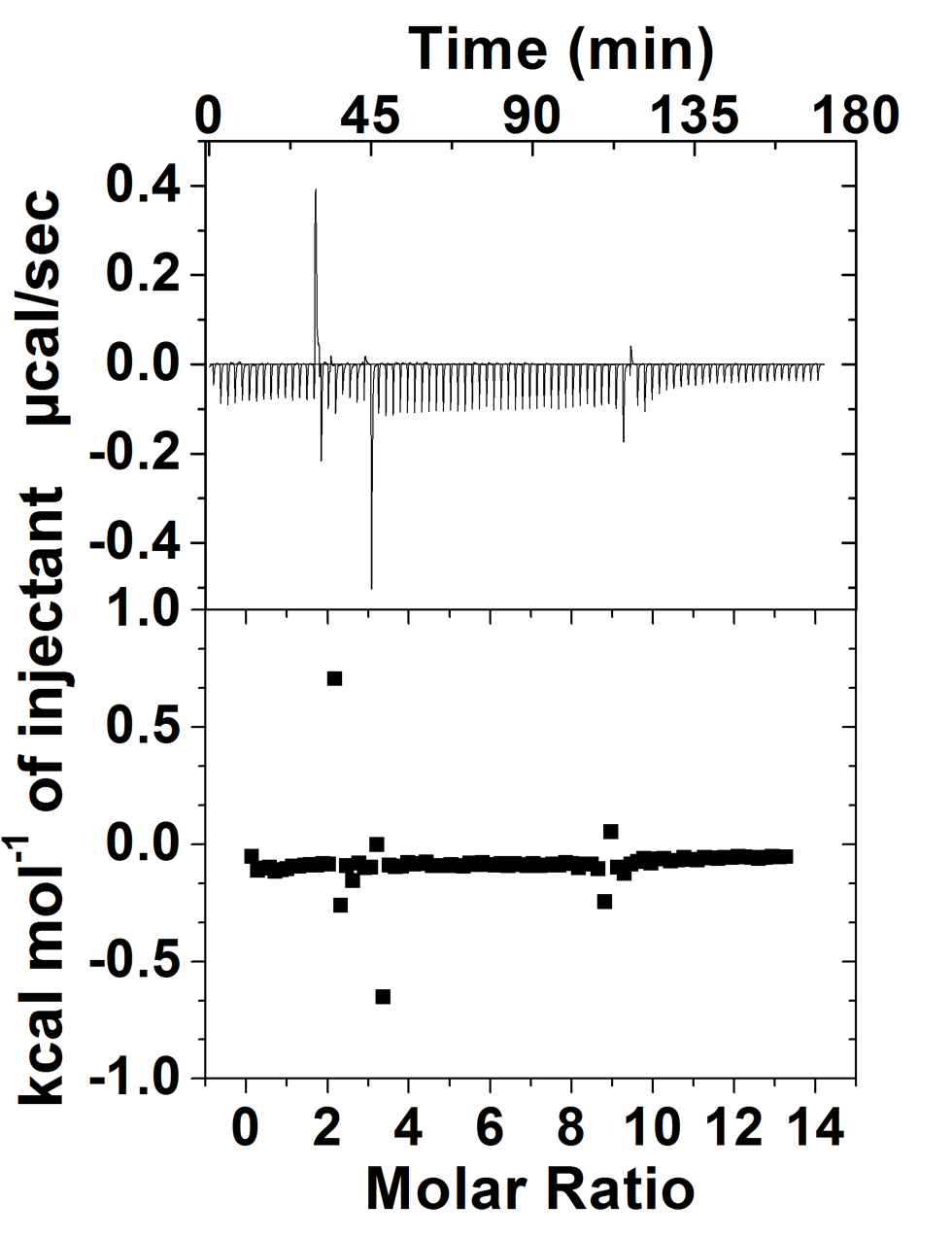

Supplement: Figure S4 — Mg2+-binding to vibrillin by ITC: The buffer used for preparation of ligand and protein for ITC experiment was 50 mM Tris-Cl (pH 7.0), 100 mM KCl. The ITC experiment was performed under the similar conditions as for Ca2+-binding. Concentrations of protein and Mg2+ used in ITC experiments were 100 µM and 10 mM respectively. The protein in a sample cell was titrated with 80 injections of 3 µl each from a stock of 10 mM MgCl2. (TIF) [file pone.0053610.s004.tif]
